# Mutation of barley *HvPDIL5-1* improves resistance to yellow mosaic virus disease without growth or yield penalties

**DOI:** 10.3389/fpls.2022.1018379

**Published:** 2022-10-06

**Authors:** Chunyuan Cheng, Jinhong Kan, Shanshan Li, Congcong Jiang, Xiaoyan He, Huiquan Shen, Rugen Xu, Boqun Li, Zongyun Feng, Ping Yang

**Affiliations:** ^1^ College of Agronomy, Qingdao Agricultural University, Qingdao, China; ^2^ Institute of Crop Sciences, Chinese Academy of Agricultural Sciences, Beijing, China; ^3^ College of Agronomy, Sichuan Agricultural University, Chengdu, China; ^4^ Institute of Agricultural Science in Jiangsu Coastal Areas, Yancheng, China; ^5^ College of Agronomy, Yangzhou University, Yangzhou, China; ^6^ Special Crops Institute, Chongqing Academy of Agricultural Sciences, Chongqing, China

**Keywords:** barley, bymovirus, BaYMV/BaMMV, genome editing, host factor, mutagenesis

## Abstract

The soil-borne yellow mosaic virus disease, which is caused by the bymoviruses barley yellow mosaic virus (BaYMV) and/or barley mild mosaic virus (BaMMV), seriously threatens winter barley production in Europe and East Asia. Both viruses are transmitted by the soil-borne plasmodiophorid *Polymyxa graminis* and are difficult to eliminate through chemical or physical measures in the field, making breeding for resistant cultivars the optimal strategy for disease control. The resistance locus *rym1/11* was cloned encoding the host factor gene *Protein Disulfide Isomerase Like 5-1* (*PDIL5-1*), whose loss-of-function variants confer broad-spectrum resistance to multiple strains of BaMMV/BaYMV. Most resistance-conferring variants have been identified in six-rowed barley landraces/historic cultivars, and their introgression into modern two-rowed malting cultivars is difficult because *PDIL5-1* is located in a peri-centromeric region with suppressed recombination. In this study, we used CRISPR/Cas9 genome editing to modify *PDIL5-1* in the BaYMV/BaMMV-susceptible elite malting barley cv. ‘Golden Promise’ and obtained the mutants *pdil5-1-a* and *pdil5-1-b*. *PDIL5-1* in the *pdil5-1-a* mutant encodes a protein lacking a cysteine residue, and *pdil5-1-b* contains a protein-coding frameshift. Both mutants were completely resistant to BaYMV. The knockout mutant *pdil5-1-b* showed complete BaMMV resistance, while *pdil5-1-a* showed decreased viral accumulation but no disease symptoms if compared to ‘Golden Promise’. Both *PDIL5-1* edited lines, as well as the previously produced EMS-induced *pdil5-1* mutant ‘10253-1-5’ in the elite malting barley cv. ‘Barke’ background, displayed no growth or yield penalties in garden experiments or bymovirus-free field trials. Line ‘10253-1-5’ showed improved resistance and yield performance compared to the wild-type and its sibling line when grown in infectious fields. Therefore, genome editing of the host factor gene *PDIL5-1* could facilitate the breeding of barley varieties with resistance to bymoviruses.

## Introduction

Barley (*Hordeum vulgare* L.; 2*n* = 14), one of the world’s oldest crops, was domesticated in the Fertile Crescent approximately 10,000 years ago (Badr et al., 2000) and currently ranks among the four most important cereal crops in yield and cultivation area worldwide (FAO dataset, 2020). About 70% of globally produced barley grain is used for animal feed, and the remainder is used by the malting/brewing industries (ca. 20%) or as human food (ca. 5%), primarily as a staple in the Himalayas and some African countries (Grando and Macpherson, 2005). Because barley is a completely inbred diploid species with a relatively small genome (ca. 5.1 Gb), as well as abundant genetically diverse germplasm stocks and genomic resources (Milner et al., 2019; Mascher et al., 2021), this plant serves as a genetic model for other crops in the Triticeae tribe (Stein and Muehlbauer, 2018).

Barley yellow mosaic virus disease, which is caused by independent or combined infection by barley yellow mosaic virus (BaYMV) and barley mild mosaic virus (BaMMV), causes severe yield losses in winter barley in Europe and East Asia (Kühne, 2009; Jiang et al., 2020). Both viruses belong to the genus *Bymovirus* of the family *Potyviridae* and are transmitted by the soil-borne plasmodiophorid *Polymyxa graminis* (Kühne, 2009). This obligate intercellular parasite, which is distributed worldwide, primarily multiplies in the roots of cereal species and forms thick-walled resting spores to protect viruses against unfavorable environments (Kanyuka et al., 2003). Once soil-borne bymoviruses occur, they become a severe, persistent threat in barley growing fields (Braselton, 1995; Bragard et al., 2013). Under natural conditions, disease symptoms become visible in early spring on the newly emerging leaves of autumn-sown susceptible varieties. The infected plants show yellow discoloration on young leaves at the vegetative growth phase, stunted growth at the jointing stage, and/or delayed maturation (Jiang et al., 2020). Although various disease-control measures are helpful in controlling outbreaks of barley yellow mosaic virus disease, including crop rotation and fungicide application, the most promising strategy is to breed varieties with viral resistance (Miedaner and Korzun, 2012; Rotenberg et al., 2016).

Plant viruses have relatively simple genomes that encode few proteins. Thus, viruses are highly dependent on host-encoded virus-compatible proteins known as susceptibility factors (*S* genes), or host factors, to fulfil their lifecycles (Whitham and Wang, 2004). Modification of host factors results in the loss of susceptibility, thereby conferring recessive (passive) resistance (Kang et al., 2005). This type of resistance allows the host to escape from viral infection and often functions differently from resistance (*R*)-gene-mediated dominant resistance (Dodds and Rathjen, 2010). To date, 22 resistance genes against BaYMV/BaMMV have been genetically identified in cultivated barley and its wild relative *Hordeum bulbosum*, most of which are recessively inherited (Jiang et al., 2020; Wang et al., 2022). Two resistance genes have been cloned, including a loss-of-function variant of the host factor gene *Protein Disulfide Isomerase Like 5-1* (*PDIL5-1*) at the *rym1/11* locus (Yang et al., 2014). Non-functional variants of barley *PDIL5-1* confer broad-spectrum, durable resistance to multiple *Bymovirus* strains, but the functional mechanism underlying this resistance remains elusive. Resistant variants of *PDIL5-1* have been observed in landraces/historic varieties in East Asia but are rarely detected in modern barley varieties, including two-rowed malting varieties (Yang et al., 2017).

Genome editing enables precise modifications to be created at the target locus to confer a desired trait, representing a next-generation strategy for plant breeding (Chen et al., 2019). CRISPR (clustered regularly interspaced short palindromic repeats)/Cas9 (CRISPR-associated protein 9) is currently the most widely used genome-editing system due to its simplicity and flexibility of vector construction, high efficiency and accuracy, and ease of use (Manghwar et al., 2019; Gao, 2021). This technology has been successfully used in many cereal crops, including wheat, rice, and maize, to improve yield or grain quality (Fiaz et al., 2019; Liu et al., 2021; Zhang et al., 2021), enhance abiotic stress tolerance (Anwar and Kim, 2020; Zafar et al., 2020; Bhat et al., 2021), and enhance disease resistance (Langner et al., 2018; Yin and Qiu, 2019; Ahmad et al., 2020; Tyagi et al., 2021; Khan et al., 2022).

Here, we precisely edited the host factor gene *HvPDIL5-1* in elite two-rowed malting barley cultivar (cv.) ‘Golden Promise’ *via* CRISPR/Cas9-mediated genome editing. The *HvPDIL5-1* knockout mutant *pdil5-1-b* showed complete resistance against BaYMV and BaMMV, whereas *pdil5-1*-*a* mutant produces a protein lacking a cysteine residue and showed complete BaYMV resistance but partial BaMMV resistance. These *pdil5-1* mutants, as well as a previously generated ethyl methanesulfonate (EMS)-induced mutant, displayed no developmental defects or yield penalties in garden experiments or field trials. Importantly, the *pdil5-1* mutants showed improved viral resistance and yield performance in disease-prevalent fields. Therefore, *HvPDIL5-1* represents a promising target for genome editing in elite barley varieties to improve bymovirus resistance.

## Materials and methods

### Vector construction

The web-based tool CRISPR-P 2.0 was used to design highly specific target sequences for CRISPR/Cas9-mediated editing; target sequences following the 5’N_20_NGG3’ rule was identified based on the *HvPDIL5-1* coding sequence in ‘Golden Promise’ (Schreiber et al., 2020). The destination vector was *pWMB110*-*SpCas9* (Liu et al., 2020), which contains the CRISPR/Cas9 cassette driven by the maize *Ubiquitin* promoter and the *bar* gene as a selection marker for the regenerated plants. A gRNA expression cassette driven by the wheat *U3* promoter was inserted into pWMB110-SpCas9 using an In-Fusion Cloning Kit (Takara Bio Inc., Japan), and this was then transformed into *Agrobacterium* strain C58C1 through tri-parental mating (Ditta et al., 1980). Primers used for plasmid construction are listed in **Supplementary Table S1**.

### *Agrobacterium*-mediated barley transformation

The European two-rowed spring barley cultivar ‘Golden Promise’ was used for *Agrobacterium*-mediated transformation. The plants were cultured in pots (18-cm diameter, one plant per pot) in a growth chamber (23°C, 16 h light/18°C, 8 h dark with 300 μmol/m^2^/s light intensity and 45% relative humidity). At 14 days post-anthesis, immature embryos 1.5-2 mm in diameter were isolated and sterilized for *Agrobacterium*-mediated transformation using a modified barley transformation protocol (Harwood, 2014). In brief, isolated immature embryos were incubated for 2 days with *Agrobacterium* harboring the CRISPR/Cas9 vectors at 23°C in the dark. The embryo axis was then excised and cultured at 25°C in the dark for 5 days, scutellum side up, on medium containing 250 mg/L carbenicillin (PhytoTech, Lenexa, KS, USA) and 100 mg/L cefotaxime (Amresco Inc., USA), and then cultured on callus induction medium containing 10 mg/L phosphinothricin to select transgenic plants containing the *bar* gene. Proliferated calli were transferred to regeneration medium and cultured for 2 weeks at 25°C under constant illumination at 100 μmol/m^2^/s. The regenerated shoots were transferred to rooting medium, and seedlings with well-developed roots were transplanted to culture soil for further growth in a growth chamber or greenhouse until harvest.

### Genotyping and Sanger sequencing

Genomic DNA was isolated from newly emerged leaves of regenerated plants (Shi et al., 2019). For T_0_ generation plants, specific primers targeting the *HvPDIL5-1* fragment containing the target site were used for PCR amplification (**Supplementary Table S1**). The purified PCR products were cloned following a standard protocol (Kan et al., 2022), and the plasmids of 8 to 10 positive colonies were sequenced by Sanger sequencing. To genotype edited T_1_ plants and their progenies containing identified events, the purified PCR products were subjected to Sanger sequencing. Immature embryos at ~15 days after anthesis were aseptically isolated and placed on half-strength MS medium for embryo culture and germination. One-week-old seedlings from the germinated embryos were transplanted to soil, and their leaves were harvested for DNA extraction and genotyping (Abe et al., 2020).

### Detection of the off-target events

To detect the off-target events, the target site was checked through BLASTN against the reference genome sequence of barley (Morex_V3; Mascher et al., 2021), and four putative off-target sites showing levels of similarity to the target sequence were predicted. Sequence-specific PCR amplification and Sanger sequencing was used to determine the potential off-target effects in both edited mutants. The primer sets were listed in **Supplementary Table S1**.

### Evaluation of virus resistance in climate-controlled chambers

Tests for resistance were conducted following mechanical inoculation under growth chamber conditions (12°C, 10 h day/8°C, 14 h night) (Shi et al., 2019). The BaYMV- or BaMMV-infected leaves were originally collected from Dazhong National Farm or Nanyang Field Trial Station in Yancheng city, Jiangsu Province, respectively. Two-week-old seedlings were mechanically inoculated using sap from virus-infected leaves mixed with K_2_HPO_4_ buffer (1:10; 0.1 M; pH 9.1) and silicon carbide (carborundum, mesh 150–200, 0.5 g/25 mL sap). A booster inoculation was performed following the same procedure 5 days after the first inoculation. Ten to twenty plants per genotype were tested in each experiment. At 6 weeks after the first inoculation, newly emerged leaves were sampled for total RNA extraction using TRIzol reagent (Invitrogen, USA), followed by first-strand cDNA synthesis using HiScript III RT SuperMix (Vazyme Biotech, Nanjing, China). Quantitative reverse-transcription PCR (qRT-PCR) was performed with ChamQ Universal SYBR qPCR Master mix (Vazyme Biotech, Nanjing, China) in an ABI 7500 Real-Time PCR system (Applied Biosystems, USA). Relative gene expression levels were calculated using the 2^-ΔΔCT^ method (Livak and Schmittgen, 2001). The primers for the barley *actin* gene (endogenous control), BaYMV, and BaMMV are listed in **Supplementary Table S1**.

### Evaluation of agronomic performance in a garden experiment

For the garden experiment, the *PDIL5-1* edited lines and wild-type ‘Golden Promise’ were sown in pots containing soil and grown under bymovirus-free conditions in the spring of 2022 to evaluate their agronomic performance throughout the plant lifecycle. Twelve agronomic traits were evaluated, including plant height (PH), days to heading (HD), days to maturation (MD), tiller number (TL), spike number per plant (SNP), grain number per main spike (GNS), spike length (SL), grain weight per plant (GWP), grain length (GL), grain width (GW), the ratio of grain length to width (RLW), and thousand-grain weight (TGW), using 10 to 15 plants of each genotype. Grain traits including GL, GW, RLW, and TGW were detected with the WSEEN (SC-G, Wseen Detection Tech, Hangzhou, China) measuring system. Ten plants per genotype were analyzed.

### Field trials

Field trials were conducted at six locations (bymovirus-free: Beijing, Chongqing, Kunming city in Yunnan province, and Xinxiang city in Henan province; virus-prevalent: Yancheng city and Yangzhou city, Jiangsu province, disease nursery). The tested genotypes were sown in October and harvested in early summer of the next year. Three replicates per trial were utilized, with a randomized block design. Within a replicate, approximately 300 kernels per genotype were sown in four rows (1.5-m in length, 25 cm between rows). Seven agronomic traits were investigated: plant height (PH), spike length (SL), grain number per spike (GNS), thousand-grain weight (TGW), the ratio of grain length to width (RLW), grain length (GL), and grain width (GW). The agronomic traits of 10 to 15 representative plants per replicate were measured, except for TGW, for which three replicates were utilized.

## Results

### Generation of barley *pdil5-1* mutants by CRISPR/Cas9-mediated genome editing

**Figure 1:**
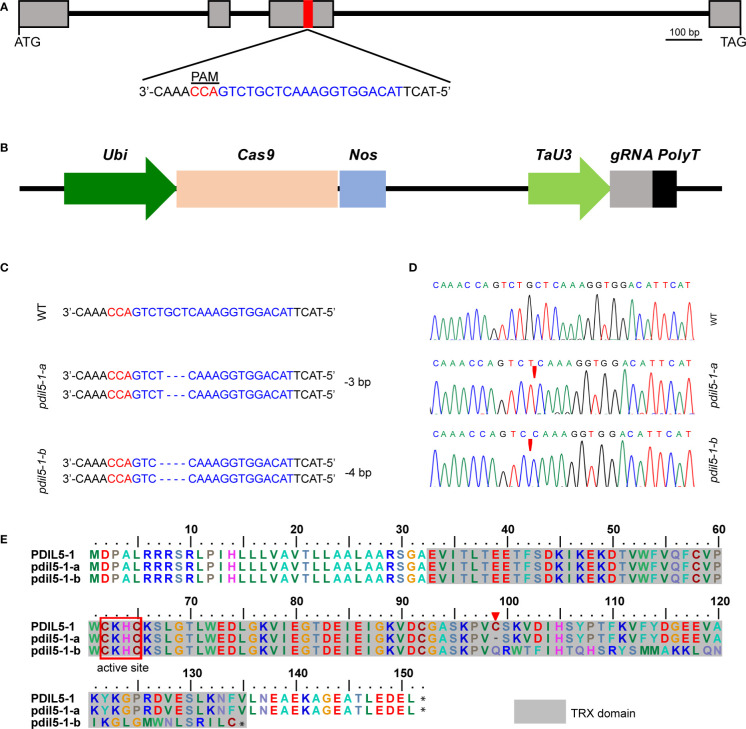
CRISPR/Cas9-induced targeted mutagenesis of *HvPDIL5-1* in barley. **(A)** Gene structure of *HvPDIL5-1* and sequence of the target site. The target site for sgRNA within the third exon of *HvPDIL5-1* is highlighted in red. The protospacer-adjacent motif (PAM) and target sequence are highlighted in red and blue, respectively. Exons and introns are shown as gray boxes and black lines, respectively. **(B)** Schematic representation of the linearized CRISPR/Cas9 vector. *Ubi*, *Zea mays Ubiquitin* promoter; *Nos*, *Nos* terminator; *TaU3*, sgRNA promoter; *PolyT*, a 7-bp poly(T) sequence. **(C)** Changes in the *HvPDIL5-1* nucleotide sequence in the two mutants. The sgRNA and PAM sequences are highlighted in blue and red, respectively. WT: wild-type sequence. ‘-’, deletion of the respective nucleotide. **(D)** Sanger sequencing chromatograms of the target region in wild type ‘Golden Promise’ and the two mutants. Red triangles represent the mutations. **(E)** Amino acid sequence alignment of PDIL5-1 in the wild type and mutants. The TRX (thioredoxin-like) domain and active center are indicated by gray and red boxes, respectively. The red triangle indicates the amino acid deletion.

*HvPDIL5-1* (HORVU.MOREX.r3.4HG0385260.1) encodes a disulfide isomerase-like 5-1 protein in barley. The non-functional variants of this gene were identified as the causal agent of naturally occurring resistance to multiple *Bymovirus* strains (Yang et al., 2014). The resistance-conferring variants were identified in various six-rowed landraces or historic cultivars of barley but are rarely detected in modern two-rowed malting cultivars (Yang et al., 2014; Yang et al., 2017). Here, to generate BaMMV/BaYMV-resistant variants in malting barley, we deployed CRISPR/Cas9 technology to edit *HvPDIL5-1* by designing one single guide RNA (sgRNA) targeted to the third exon of this gene, within the thioredoxin (TRX) domain and upstream of the protospacer-adjacent motif (PAM; TGG) sequence (**Figure 1A**). We used a construct bearing both the sgRNA and the Cas9 cassette (**Figure 1B**) to transform immature embryos of elite two-rowed malting barley cv. ‘Golden Promise’ *via Agrobacterium*-mediated transformation. Ninety-seven regenerated T_0_ plants were identified. Among these, we detected nine editing events, representing an editing efficiency of 9.3% (**Table 1**); 44.4% of the mutant plants were heterozygous, 33.3% were bi-allelic, 11.1% were homozygous, and 11.1% were chimeric mutants. Most editing events were either a 4-bp deletion leading to a frameshift mutation or a 3-bp deletion resulting in an amino acid deletion. Six T_0_ plants with either a 4-bp or a 3-bp deletion successfully set seed (**Table 1**).

**Table 1:** The mutations detected in 97 regenerated T_0_ plants by Sanger sequencing of plasmids derived from PCR products.

No.	Lines	Mutations	Denotation	Note
1	1	−4 bp, −4 bp	bi-a	normal seeds
2	4	−4 bp, −4 bp,	bi-a	normal seeds
3	15	−4 bp, −4 bp	bi-a	normal seeds
4	20	+1 bp, wt	het	no seeds
5	53	−3 bp, −4 bp, wt	chi	normal seeds
6	55	−32 bp	hom	no seeds
7	66	−3 bp, wt	het	no seeds
8	96	−3 bp, wt	het	normal seeds
9	97	−3 bp, wt	het	normal seeds

het, heterozygote; hom, homozygote; chi, chimeric; bi-a, bi-allelic; wt, wild−type.

We chose three edited lines (#1, #4, #53) for seed propagation. The progenies of lines #1 and #4 harbored a 4-bp deletion mutation in a bi-allelic pattern, like that of their parents, but the plantlets failed to produce seeds. The T_1_ progeny of line #53 included both wild-type and mutant plants, including plants with a homozygous 3-bp deletion, a homozygous 4-bp deletion, and both deletions (**Supplementary Table S2**). Since the homozygous T_1_ mutant plants produced few seeds, we genotyped seeds of two heterozygous T_1_ plants (#53-1 and #53-2) and propagated the plants for one generation to obtain homozygous T_3_ mutants. The homozygous T_3_ mutants with the 3-bp and the 4-bp deletion were designated as *pdil5-1-a* and *pdil5-1-b*, respectively (**Figures 1C, D**). The *pdil5-1-a* mutant harbored a deletion of a conserved amino acid (cysteine, C_99_), and the *pdil5-1-b* mutant harbored a coding frameshift within the TRX domain (**Figure 1E**). The CRISPR/Cas9 cassette was present in both mutants (**Supplementary Figure S1**). We also assessed the potential off-target effects in the edited plants, and predicted four putative off-targets sites of sgRNA according to the barley reference genome sequence (Morex_V3). By sequence-specific PCR amplification and Sanger sequencing of the potential off-target sites, no mutations were detected in both edited mutant lines (**Supplementary Table S3**), indicating off-targets unlikely occurred.

### Mutation of *HvPDIL5-1* confers resistance to BaMMV and BaYMV

**Figure 2:**
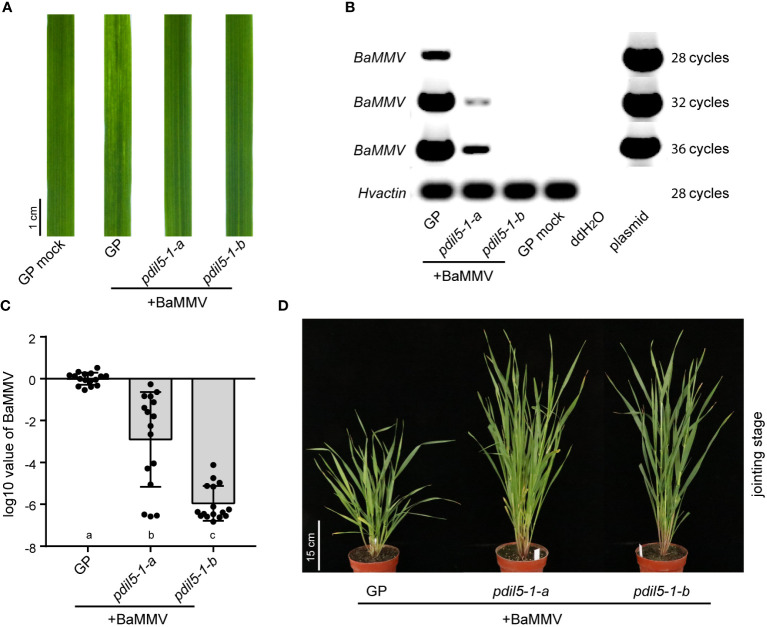
Editing of *HvPDIL5-1* improves BaMMV resistance. **(A)** Phenotypes of the *pdil5-1* mutants and wild type (GP, Golden Promise) at 6 weeks post BaMMV inoculation. GP mock, Golden Promise inoculated with buffer only. RT-PCR **(B)** and qRT-PCR **(C)** detection of BaMMV accumulation. The barley *actin* gene was used as the endogenous control. In **(C)**, black dots represent individual assays (*n* = 16). Statistical tests were conducted using Tukey’s *post hoc* analysis of variance (ANOVA) (*p* = 0.05); different letters below bars indicate statistically significant differences. Error bars represent standard deviation (SD). **(D)** Phenotypes of BaMMV-inoculated *PDIL5-1-*edited mutant lines at the jointing stage under normal greenhouse conditions.

To evaluate whether the mutation of *HvPDIL5-1* modified the response of barley to viral infection, we mechanically inoculated the two homozygous mutants (*pdil5-1*-a and *pdil5-1-b*) and wild-type ‘Golden Promise’ with BaMMV-infected barley leaves (isolate, BaMMV_CN_NY; NCBI accession ID, MW295878) (Jiang et al., 2022) collected from Nanyang Field Trial Station in Yancheng, China, or performed mock inoculation (buffer only). All mock-inoculated plants stayed green and grew well. When inoculated with BaMMV, both *padil5-1-a* and *padil5-1-b* showed green leaves (no disease symptoms), whereas wild-type barley showed typical yellow discoloration on newly emerging leaves (**Figure 2A**).

We performed reverse transcription (RT)-PCR and quantitative RT-PCR (qRT-PCR) to quantify BaMMV accumulation. Viral RNA accumulation was barely detected in *pdil5-1-b* (ca. 10^-6^) or *pdil5-1-a* (ca. 10^-3^) plants, whereas abundant viral RNA accumulation was detected in the susceptible wild-type ‘Golden Promise’ (**Figures 2B, C**). The *pdil5-1-a* plants showed decreased BaMMV accumulation compared to the wild type, indicating that the deletion of amino acid Cys-99 suppressed BaMMV proliferation in the leaves. The knock-out mutant *pdil5-1-b* showed complete resistance to BaMMV (no viral RNA detected). We transferred 8-week-old plants that were inoculated with BaMMV at the seeding stage to a greenhouse under normal conditions. Inoculated ‘Golden Promise’ plants showed severe dwarfing at the jointing stage, whereas *pdil5-1-a* and *pdil5-1-b* plants were taller and grew well (**Figure 2D**).

**Figure 3:**
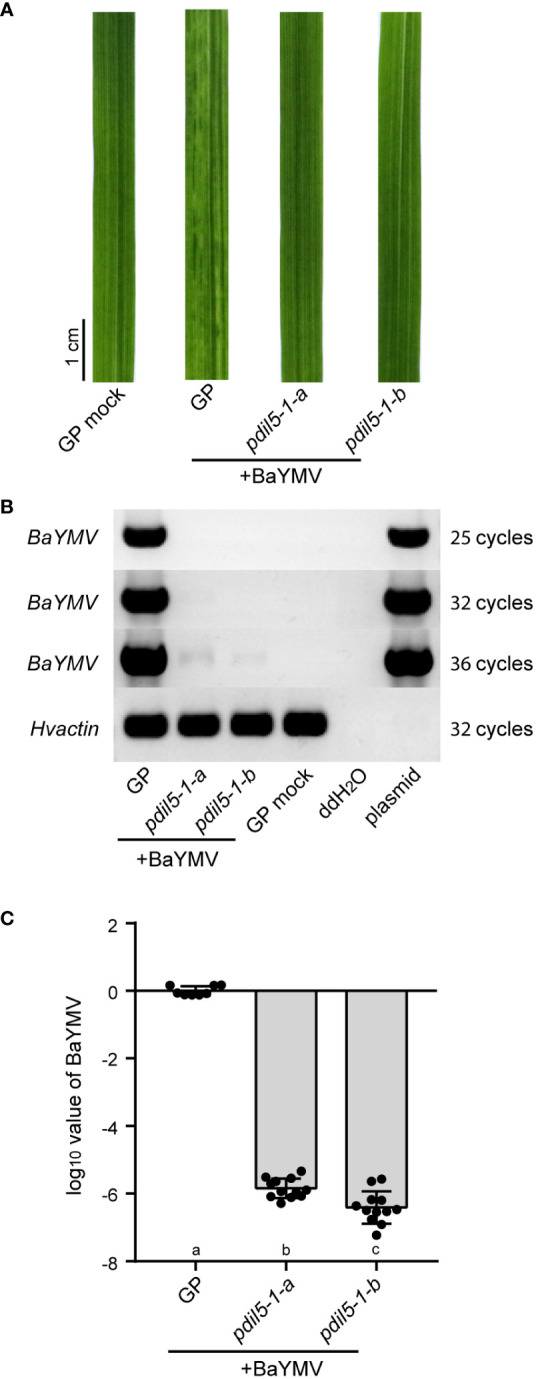
The *pdil5-1* mutants confer BaYMV resistance. **(A)** Disease symptoms of the *pdil5-1* mutants and wild type (GP, Golden Promise) at 6 weeks post BaYMV inoculation. RT-PCR **(B)** and qRT-PCR **(C)** detection of BaYMV accumulation. The barley *actin* gene was used at the endogenous control. In **(C)**, black dots represent individual assays (*n* = 8 for wild type; *n* = 12 for *pdil5-1* mutants). Error bars = SD. Statistical tests were conducted using Duncan’s multiple range test analysis of variance (ANOVA) (*p* = 0.05); different letters below bars indicate statistically significant differences.

We also inoculated the mutants using BaYMV-infected leaves (isolate, BaYMV_CN_DZ; NCBI accession ID, MW295871) (Jiang et al., 2022) collected from Dazhong National Farm in Yancheng city, China. Under this treatment, *padil5-1-a* and *padil5-1-b* plants had green leaves, whereas wild-type ‘Golden Promise’ plants showed susceptibility to the virus, with yellow discoloration (**Figure 3A**). RT-PCR and qRT-PCR showed little viral RNA accumulation (ca.10^-6^) in *pdil5-1-a* and *pdil5-1-b* plants, but abundant viral RNA accumulation in the susceptible wild-type plants (**Figures 3B, C**).

Taken together, these results demonstrate that editing the susceptibility factor gene *PDIL5-1* disrupted BaYMV and BaMMV infection, resulting in improved bymovirus resistance.

### The agronomic performance of *HvPDIL5-1*-edited mutants in a garden experiment

**Figure 4:**
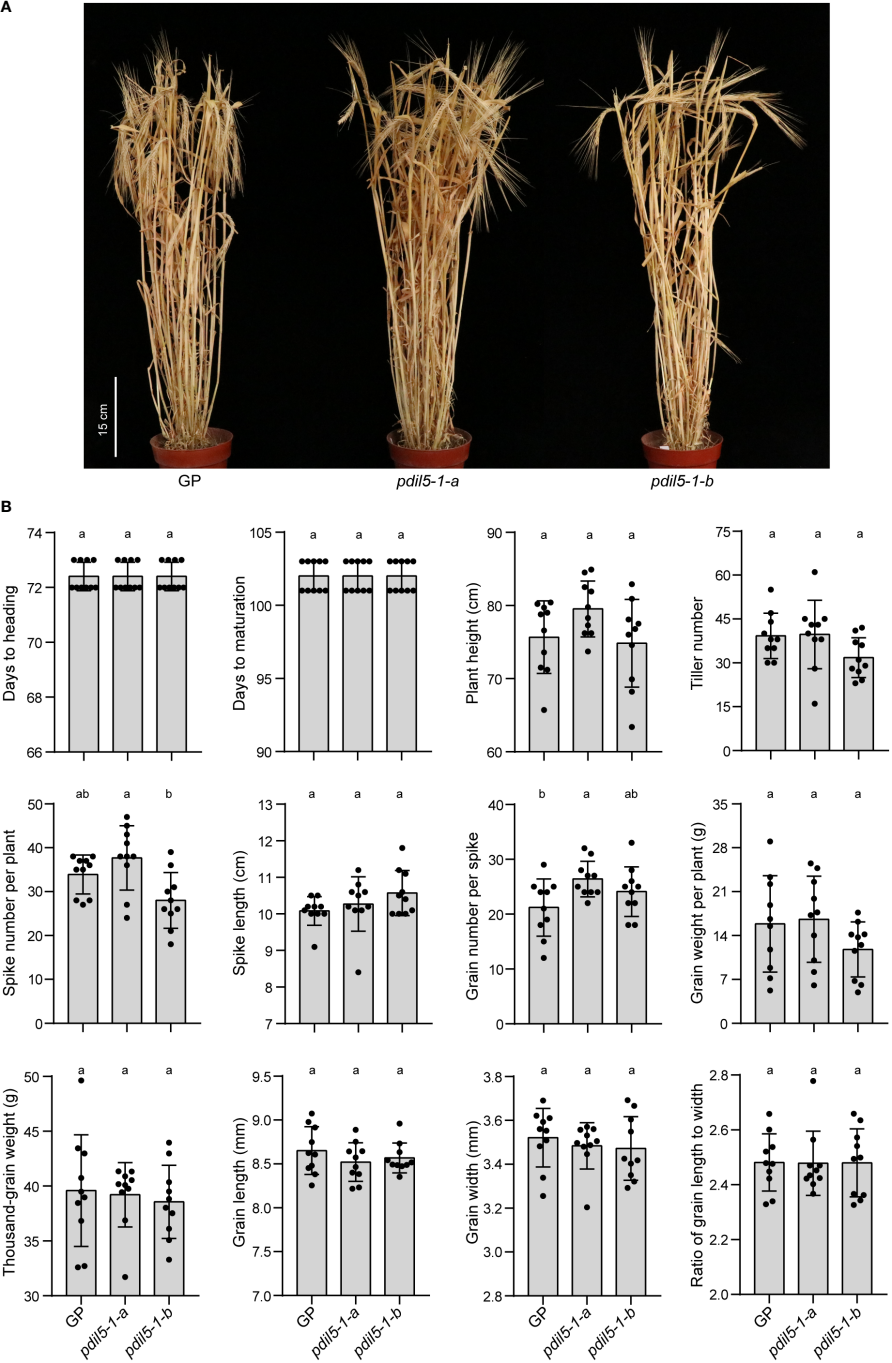
Agronomic performance of the *HvPDIL5-1*-edited mutants under normal conditions in a garden experiment. **(A)** Wild type (GP) and *pdil5-1* plants at the maturity stage. **(B)** Assessment of wild type and *pdil5-1* plants. Statistical analysis was performed using Tukey’s *post hoc* ANOVA (*p* < 0.05); different letters on bars indicate statistically significant differences. Ten plants per genotype were analyzed, and black dots represent individual measurements. Error bars = SD.

To identify whether editing barley *PDIL5-1* would cause pleiotropic effects, we investigated the agronomic performance of genetically modified *PDIL5-1*-edited plants under normal growth conditions throughout the plant lifecycle by performing a garden experiment (**Figure 4**). Overall, we found no significant difference between *PDIL5-1*-edited and wild-type plants for 12 agronomic traits: days to heading (HD), days to maturation (MD), plant height (PH), tiller number (TN), spike number per plant (SNP), spike length (SL), grain number per spike (GNS), grain weight per plant (GWP), thousand-grain weight (TGW), grain length (GL), grain width (GW), and the ratio of grain length to width (RLW) (**Figure 4B**). We also observed differences among the two mutants and the wild-type plants in two traits: higher spike number per plant (SNP) in the *pdil5-1-a* mutant compared to the *pdil5-1-b* mutant, and increased grain number per spike (GNS) in *pdil5-1-a* vs. wild-type ‘Golden Promise’. These results indicate that *PDIL5-1* is a desirable target for engineering bymovirus resistance without a penalty in development or yield performance.

### The performance of EMS-induced *pdil5-1* mutants in field trials

**Figure 5:**
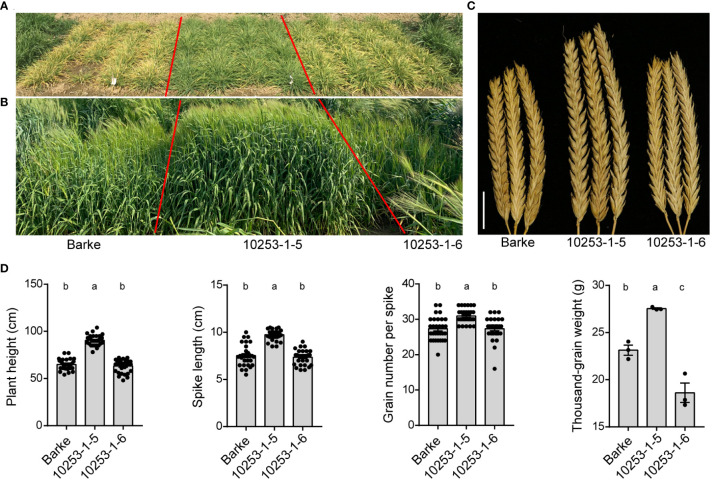
Trials of EMS-induced *pdil5-1* mutants in virus-prevalent fields. Three independent plots per genotype were analyzed. **(A)** Assessment of the *pdil5-1* mutant (10253-1-5), its sibling line (10253-1-6), and the parental line ‘Barke’ for disease resistance in a disease nursey in Yancheng, Jiangsu Province (2020-2021). The photograph was taken at the end of February at the tillering stage. **(B)** The latter two genotypes show stunted growth and fewer tillers at the heading stage compared to the *pdil5-1* mutant. **(C)** The spikes of plants grown in virus-prevalent fields. Scale bar = 2 cm. **(D)** Statistics of the agronomic performance of four traits. Statistical analysis was performed using Tukey’s *post hoc* ANOVA (*p* < 0.05); different letters on bars indicate statistically significant differences. For plant height, spike length, and grain number per spike, 30 plants per genotype were analyzed. Thousand-grain weight was measured based on the performance of three plots per genotype. Error bars = SD.

To investigate the performance of *pdil5-1* mutants in field trials with and without virus prevalence, we used a previously generated homozygous mutant, ‘10253-1-5’, carrying a premature stop codon in the coding sequence (CDS; G183A) of *HvPDIL5-1*, along with its wild-type sibling line ‘10253-1-6’, carrying a homozygous functional *HvPDIL5-1*. Both lines were identified by targeting induced local lesions in genomes (TILLING) screening of an EMS-induced mutant population derived from barley cv. ‘Barke’ (Gottwald et al., 2009; Yang et al., 2014), followed by propagation through several generations to obtain M_8_ seeds. ‘Barke’ is a European two-rowed malting barley that is susceptible to both BaYMV and BaMMV. The ‘10253-1-5’ mutant is completely resistant to BaMMV/BaYMV, whereas ‘10253-1-6’ is susceptible to these viruses (Yang et al., 2014). We conducted plot trials in eight environments, including BaMMV/BaYMV-prevalent fields (Yancheng and Yangzhou, China) and standard fields (Beijing, Chongqing, Kunming and Xinxiang, China) from 2019 to 2021 (**Table 2**). In the BaMMV/BaYMV-prevalent fields, such as in Yancheng in the early spring of 2020, the *pdil5-1* mutant ‘10253-1-5’ was resistant to bymoviruses and grew well compared to the susceptible parental ‘Barke’ and its wild-type sibling line ‘10253-1-6’. The latter two genotypes showed chlorosis of leaves at the tillering stage (**Figure 5A**), dwarfism at the heading stage (**Figure 5B**), and decreased plant height (PH), spike length (SL), spike number per plant (SNP), and thousand-grain weight (TGW) (**Figures 5C, D**, **Table 2**). No significant differences in seven agronomic traits were observed between *pdil5-1* mutant and its sibling line or wild-type ‘Barke’ in BaMMV/BaYMV-free fields (**Table 2**). These results demonstrate that a non-functional mutation of *HvPDIL5-1* could prevent yield losses without obvious growth differences or yield penalty in infectious fields.

**Table 2:** Field performance of ‘Barke’, the EMS-induced *pdil5-1* mutant (10253-1-5), and its non-mutated sibling line (10253-1-6) in six locations in three years.

Environment	Genotype	PH (cm)	SL (cm)	GNS	TGW (g)	RLW	GL (mm)	GW (mm)
2019_Beijing	Barke	70.6 ± 3.4	11.7 ± 0.8	26.5 ± 3.3	50.7 ± 0.4	2.4 ± 0.1	9.4 ± 0.1	3.9 ± 0.1
	10253-1-5	70.8 ± 8.3	10.6 ± 1.1	27.7 ± 3.6	51.9 ± 0.8	2.4 ± 0.1	9.3 ± 0.1	3.9 ± 0.1
	10253-1-6	71.8 ± 4.3	10.2 ± 0.9^*^	27.7 ± 2.6	50.3 ± 0.1	2.3 ± 0.1	9.1 ± 0.1^*^	3.9 ± 0.1
2020_Beijing	Barke	84.4 ± 8.7	9.1 ± 1.4	25.1 ± 3.3	36.4 ± 2.9	2.5 ± 0.03	8.1 ± 0.3	3.3 ± 0.1
	10253-1-5	80 ± 7.2	9.2 ± 0.9	23.4 ± 2.9	37.8 ± 4.4	2.5 ± 0.03	8.3 ± 0.3	3.3 ± 0.1
	10253-1-6	83.3 ± 9.1	9.1 ± 1.0	23.6 ± 3.8	38.7 ± 2.5	2.5 ± 0.08	8.2 ± 0.5	3.4 ± 0.1
2020_Chongqing	Barke	75.7 ± 1.7	8.1 ± 0.8	27.3 ± 3.5	37.4 ± 3.0	2.2 ± 0.1	7.5 ± 0.1	3.5 ± 0.1
	10253-1-5	66.5 ± 9.1	7.1 ± 1^**^	23.5 ± 3.9^**^	36.1 ± 1.6	2.2 ± 0.1	7.6 ± 0.2	3.4 ± 0.1
	10253-1-6	70.5 ± 12.7	6.4 ± 0.9	21.1 ± 3.5	33.2 ± 1.1	2.2 ± 0.1	7.2 ± 0.1	3.3 ± 0.1
2021_Kunming	Barke	53.2 ± 3.2	8.2 ± 0.6	26.9 ± 2.7	NA	NA	NA	NA
	10253-1-5	53.2 ± 4.8	8.5 ± 1.0	27.6 ± 2.8	NA	NA	NA	NA
	10253-1-6	51.1 ± 3.3	8.1 ± 1.1	25.0 ± 3.7	NA	NA	NA	NA
2021_Xinxiang	Barke	95.9 ± 4.1	8.3 ± 0.6	29.2 ± 2.7	NA	NA	NA	NA
	10253-1-5	96.8 ± 4.4	8.4 ± 0.9	29.2 ± 2.1	NA	NA	NA	NA
	10253-1-6	94.3 ± 3.4	8.4 ± 0.9	28.7 ± 2.0	NA	NA	NA	NA
2020_Yangzhou (Virus infected)	Barke	69.2 ± 8.5	7.9 ± 1	28.4 ± 3.2	36.6 ± 9	2.7 ± 0.2	8.6 ± 0.2	3.3 ± 0.3
	10253-1-5	81.9 ± 11.1^**^	9.6 ± 1^**^	31 ± 2.8^*^	41.4 ± 0.7	2.5 ± 0.1	8.6 ± 0.1	3.5 ± 0.1
	10253-1-6	66.2 ± 7.6	8.4 ± 0.7	27.6 ± 3.8	41 ± 1.7	2.6 ± 0.1	8.8 ± 0.1	3.4 ± 0.1
2020_Yencheng (Virus infected)	Barke	64.9 ± 5.6	7.5 ± 1.1	27.3 ± 3.2	23.1 ± 0.8	2.8 ± 0.1	8 ± 0.2	2.9 ± 0.1
	10253-1-5	90.6 ± 5.5^**^	9.7 ± 0.6^**^	30.9 ± 1.9^**^	27.5 ± 0.1^*^	2.5 ± 0.1	7.7 ± 0.4	3.0 ± 0.1
	10253-1-6	63.4 ± 6.7	7.4 ± 0.8	27.3 ± 3.2	18.6 ± 1.5^*^	3 ± 0.1	8.1 ± 0.2	2.8 ± 0.1
2021_Yencheng (Virus infected)	Barke	69.6 ± 7.3	9.5 ± 0.9	28.3 ± 2.8	NA	NA	NA	NA
	10253-1-5	80.2 ± 6.5^**^	8.9 ± 1.0	28.6 ± 1.8	NA	NA	NA	NA
	10253-1-6	71.9 ± 6.9	9.7 ± 0.9	26.8 ± 2.6	NA	NA	NA	NA

PH, plant height; SL, spike length; GNS, grain number per spike; TGW, thousand-grain weight; RLW, the ratio of grain length to width; GL, grain length; GW, grain width. The values are represented with mean± standard deviation (SD). n = 10 to 30, except for GL, GW and RLW (n = 100). The pairwise statistic tests (mutant/sibling vs. ‘Barke’) were conducted using the two-tailed Student’s t-test, ^*^Significant at p< 0.05. ^**^Significant at p < 0.001. NA, not analyzed.

## Discussion

Barley yellow mosaic disease, which is caused by BaYMV and BaMMV, and transmitted by soil-borne *P. graminis*, significantly threatens winter barley production in large areas of Europe and East Asia (Kühne, 2009; Jiang et al., 2022). The deployment of agricultural machinery further accelerates the spread of virus-contaminated soils. The breeding of new resistant barley varieties represents the most efficient and promising strategy to control this disease. The recessive resistance locus *rym1/11* was previously isolated through map-based cloning; its wild-type haplotype *HvPDIL5-1* is a susceptibility factor that facilitates viral proliferation (Yang et al., 2014). Loss-of-function variants of *HvPDIL5-1* have been detected in historic East Asian cultivars, especially six-rowed winter barley accessions (Yang et al., 2014; Yang et al., 2017). However, these resistance variants are not found in modern Chinese two-rowed malting varieties that were developed from European or American barley varieties, showing an apparent genomic scaffold to the traditional Eastern barley. In addition, *rym1/11* is located near a centromeric region, leading to suppressed recombination (Mascher et al., 2017), which could make it difficult to introgress this locus into malting varieties.

Yellow mosaic virus disease is the most important disease of barley in the downstream basin of Yangtze River in China. Developing two-rowed resistant malting varieties with spring or semi-winter habitats (mild winter climate) is a major breeding target in this region (Zhang and Zhang, 2003). Although isolates that overcome *rym1/11* have been reported in Japan and China, the deployment of the corresponding resistance locus provided only moderate resistance (Okada et al., 2003; Jiang et al., 2022). In this study, we obtained two *pdil5-1* knockout mutants in the European two-rowed malting barley variety ‘Golden Promise’ background through CRISPR/Cas9 genome editing. These mutants were resistant to both BaMMV and BaYMV. These two mutants, together with previously generated EMS-induced *pdil5-1* mutants in the European malting barley variety ‘Barke’ background (Yang et al., 2014), showed viral resistance in field trials. We therefore generated two resistant donor lines that could be crossed with climate-adapted malting barley varieties in order to deploy this important gene to breed BaMMV/BaYMV-resistant malting barley varieties. In addition, our findings highlight the promise of knocking out barley *HvPDIL5-1 via* genome editing in Chinese elite two-rowed malting barley varieties.

While disrupting *S* genes is a promising strategy for crop resistance breeding, *S* genes may have many essential biological functions, and their deletion might therefore cause undesired pleiotropic effects (Van Schie and Takken, 2014). In this study, we found that the barley *pdil5-1* mutant plants displayed no yield penalty or other adverse pleiotropic effects under either normal or infectious conditions, making *HvPDIL5-1* an ideal target for improving virus resistance through genome editing. Wheat *TaPDIL5-1*, an orthologous gene of barley *HvPDIL5-1*, functions as a host factor to another bymovirus, wheat yellow mosaic virus (WYMV). The simultaneous editing of its three homoeoalleles led to WYMV resistance without a detected penalty (Kan et al., 2022). By contrast, editing of other *S* genes can impair plant growth and lead to developmental defects. For example, knockout variants of the barley host factor gene *eIF4E* (*Eukaryotic Translation Initiation Factor 4E*) show decreased total grain yield per plant (Hoffie et al., 2021). The loss of function of eIF4E or its isoforms eIF(iso)4E leads to dwarfism or the loss of fertility in Arabidopsis, tobacco, tomato, and melon (Combe et al., 2005; Mazier et al., 2011; Callot and Gallois, 2014; Patrick et al., 2014; Gauffier et al., 2016; Pechar et al., 2022). Knockout of the powdery mildew susceptibility factor gene *MLO* (Buschges et al., 1997) in barley or its orthologous gene in wheat results in cell death and early senescence or growth defects in terms of both plant height and grain yield (Jørgensen, 1992; Piffanelli et al., 2002; Piffanelli et al., 2004; Wang et al., 2014). The dispensability of *PDIL5-1* in barley and wheat might be due to an unidentified compensation mechanism, as *PDIL5-1* belongs to the *PDI* gene family, which contains more than 10 members in various species (Ellgaard and Ruddock, 2005; Houston et al., 2005). *PDIL5-1* is highly conserved and was detected in most plant and animal species (Yang et al., 2014), suggesting it might function as a susceptibility factor in other species beyond wheat and barley.

The *PDI* gene family encodes PDI and PDI-like (PDIL) proteins, which are involved in catalyzing disulfide bonding formation, isomerization, and reduction/oxidation of proteins in the endoplasmic reticulum and function as chaperones to inhibit the aggregation of misfolded proteins or partially folded protein precursors (Onda, 2013; Adams et al., 2019; Urade, 2019). *HvPDIL5-1* encodes a specific PDI family member containing a single functional TRX domain whose ortholog in soybean (GmPDIL6) exhibits dithiol oxidase activity and extremely low oxidative refolding activity but lacks disulfide reductase activity (Okuda et al., 2020). In this study, we obtained the *pdil5-1-a* mutant, harboring HvPDIL5 lacking a cysteine residue. Reduced BaMMV accumulation but no disease symptoms were observed in this mutant compared to the susceptible wild-type plant. In contrast to loss-of-function variants that terminate viral multiplication and confer complete BaMMV resistance, this mutant would be useful for investigating whether PDIL5-1 facilitates intracellular viral replication or the intercellular local/systemic translocation of viruses in the future.

## Data availability statement

The original contributions presented in the study are included in the article/**Supplementary Material**. Further inquiries can be directed to the corresponding authors.

## Author contributions

PY designed research; CC, JK, SL, CJ, XH, HS, RX, BL, and ZF carried out the experiments; JK, CC, SL, and PY analyzed the results; JK and PY wrote the manuscript with inputs from all authors. All authors read and approved the final manuscript.

## Funding

This work was funded by the National Natural Science Foundation of China (32071997),Agricultural Science and Technology Innovation Program of CAAS, and Fundamental Research Funds for Central Non-Profit of Institute of Crop Sciences of CAAS.

## Acknowledgments

The authors would like to thank Drs. Xingguo Ye and Ke Wang (Chinese Academy of Agricultural Sciences) for assistance with barley transformation.

## Conflict of interest

The authors declare that the research was conducted in the absence of any commercial or financial relationships that could be construed as a potential conflict of interest.

## Publisher’s note

All claims expressed in this article are solely those of the authors and do not necessarily represent those of their affiliated organizations, or those of the publisher, the editors and the reviewers. Any product that may be evaluated in this article, or claim that may be made by its manufacturer, is not guaranteed or endorsed by the publisher.
