# Supplementary material for: Mutation of barley HvPDIL5-1 improves resistance to yellow mosaic virus disease without growth or yield penalties
Source: Front Plant Sci. 2022 Oct 6;13:1018379. doi: 10.3389/fpls.2022.1018379 (PMC9583009; doi:10.3389/fpls.2022.1018379)
Supplement: Supplementary file 1 [file DataSheet_1.pdf]

Supplementary Material

Article title: **Mutation of barley *HvPDIL5-1* improves resistance to yellow mosaic virus disease without growth or yield penalties**

There are one supplementary figure and two supplementary tables, and the following supplementary information is available for this article:

Supplementary Figure S1. Detection of the *Cas9* cassette in the *pdil5-1* mutants.

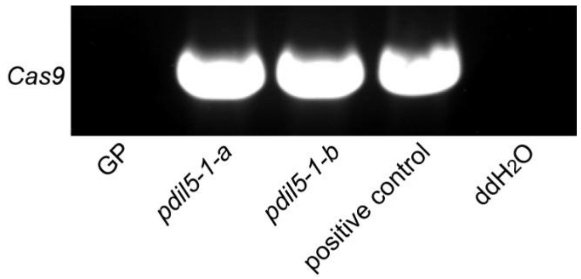

Supplementary Table S1. PCR primers used in this study.

Primer name	Sequence (5' to 3')	Experimental purpose
Cas9-F	TCAAGGCTCTTGTTCGTCAGCA	Detection of CRISPR/Cas9 cassette
Cas9-R	TTGCCGCTCTGCTTATCCCTGA	
TaU3F	GAATTCATCCTCACGTTCAACACC	Amplification of the *TaU3* promoter
TaU3R	CCTGTTCAAGTAAGGTCGTGCTTC	
HvPDIL5-1-F	CTGTATGGTTCAAGTTGCTTCGCTG	Amplification of the *HvPDIL5-1* fragment
HvPDIL5-1-R	CAACATGGTTTTCATGGTAGTACTCC	
BaYMV qPCR F	CATGCTCGAATGAATGGTGGAC	qRT-PCR of BaYMV accumulation
BaYMV qPCR R	ATACGTTCCAGTTCCAAGTGCT	
BaYMV RT F	GCAGATGGTAACTGGAGCGTG	RT-PCR of BaYMV accumulation
BaYMV RT R	TTAGGTTAGTTCTGGGTGTCCATC	
BaMMV qPCR F	TCACTCTCCTGGTTATGGTTGC	qRT-PCR of BaMMV accumulation
BaMMV qPCR R	GGTATTTCGAGCTTGTTTCCGG	
BaMMV RT F	GATTGCATACTCAGGTGGAGAG	RT-PCR of BaYMV accumulation
BaMMV RT R	CTGTATGTGCGCAGGAGCTAT	
Hvactin F	GTGTGATGTGGATATCAGGAAGG	Endogenous control (*actin* gene)
Hvactin R	TTAGAAGCACTTCCGGTGGA	
off-target1 F	CTGGATCGTGAGGAACGACCT	Detection mutations on putative off-target site of HvPDIL5-1 target
off-target1 R	CCCAGCTTAATTATTTGTTATTG	
off-target2 F	CCATGAATTGTACAGGTTGGGC	Detection mutations on putative off-target site of HvPDIL5-1 target
off-target2 R	GCCCGGAACCTTACAACTCATA	
off-target3 F	GCTTCCATGTCTTCCTGCAGG	Detection mutations on putative off-target site of HvPDIL5-1 target
off-target3 R	TTGCCCATAGTCCTCCAGAAAC	
off-target4 F	GGGAGCTGAGCCTGTATAACAA	Detection mutations on putative off-target site of HvPDIL5-1 target
off-target4 R	TGGAGTTGAAGTAGGCGAACTG	

Supplementary Table S2. The mutations detected in T_1 plants by Sanger sequencing of PCR products.

Lines	Code	Mutations	Denotation	Note
1	1-1	−4 bp, −4 bp	bi-allelic	dead
	1-2	−4 bp, −4 bp	bi-allelic	dead
	1-3	−4 bp, −4 bp	bi-allelic	dead
	1-4	−4 bp, −4 bp	bi-allelic	dead
	1-5	−4 bp, −4 bp	bi-allelic	dead
4	4-1	−4 bp, −4 bp,	bi-allelic	dead
	4-2	−4 bp, −4 bp	bi-allelic	dead
	4-5	−4 bp, −4 bp	bi-allelic	dead
	4-6	−4 bp, −4 bp	bi-allelic	dead
	4-7	−4 bp, −4 bp	bi-allelic	dead
	4-8	−4 bp, −4 bp	bi-allelic	dead
53	53-1	−3 bp, −4 bp	bi-allelic	normal seeds
	53-2	−3 bp, −4 bp	bi-allelic	normal seeds
	53-3	−3 bp	hom	fewer seeds
	53-5	−3 bp	hom	fewer seeds
	53-6	−3 bp	hom	fewer seeds
	53-8	−3 bp, −4 bp	bi-allelic	normal seeds
	53-9	−4 bp	hom	no seeds
	53-10	−3 bp, −4 bp	bi-allelic	normal seeds
	53-11	−3 bp	hom	fewer seeds
	53-12	−3 bp, −4 bp	bi-allelic	normal seeds
	53-14	−3 bp	hom	fewer seeds
	53-15	−3 bp	hom	fewer seeds
	53-16	wt	wt	normal seeds
	53-17	−3 bp, −4 bp	bi-allelic	normal seeds
	53-18	−3 bp	hom	no seeds
	53-19	−4 bp	hom	no seeds
	53-20	−3 bp, −4 bp	bi-allelic	normal seeds
	53-21	−3 bp, −4 bp	bi-allelic	normal seeds
	53-22	−3 bp, −4 bp	bi-allelic	normal seeds

hom, homozygote; bi-a, bi-allelic; wt, wild−type.

Supplementary Table S3. Analysis of potential off-target effects.

Name of putative off-target site	Putative off-target locus	Sequence of the putative off-target site	No. of mis-matching bases	Mutations
off-target1	HORVU.MOREX.r3.2HG0099640.1	CCTTGAGAGTCTGCTCAAAGATG	5	no
off-target2	HORVU.MOREX.r3.3HG0281860.1	CTACTGCCAAAAGGTGGACATGG	5	no
off-target3	HORVU.MOREX.r3.6HG0575350.1	CCTGATCTTGATCAAAGGTGGAC	6	no
off-target4	HORVU.MOREX.r3.4HG0394400.1	CTTTCAAAGGTGGACATGTCTGG	6	no

The PAM motifs are underlined and highlighted in blue, mismatching bases are shown in red.
